# Multidisciplinary consensus on optimising the detection of *NTRK* gene alterations in tumours

**DOI:** 10.1007/s12094-021-02558-0

**Published:** 2021-02-23

**Authors:** P. Garrido, R. Hladun, E. de Álava, R. Álvarez, F. Bautista, F. López-Ríos, R. Colomer, F. Rojo

**Affiliations:** 1 Sociedad Española de Oncología Médica (SEOM), Departamento de Oncología Médica, Hospital Universitario Ramón y Cajal, Universidad de Alcalá, IRYCIS, CIBERONC, Madrid, Spain; 2 Sociedad Española de Hematología y Oncologías Pediátricas (SEHOP), Departamento de Oncología, Hematología y Trasplante de Progenitores Hematopoyéticos Pediátricos, Hospital Universitario Vall d’Hebron, Barcelona, Spain; 3 Sociedad Española de Anatomía Patológica (SEAP), Departamento de Citología e Histología Normal y Patológica, Hospital Universitario Virgen del Rocío, Instituto de Biomedicina de Sevilla (IBiS), CSIC, Facultad de Medicina, Universidad de Sevilla, CIBERONC, Sevilla, Spain; 4 Sociedad Española de Oncología Médica (SEOM), Departamento de Oncología Médica, Hospital Universitario Gregorio Marañón. Instituto Investigación Sanitaria Gregorio Marañon (IISGM), Madrid, Spain; 5 Sociedad Española de Hematología y Oncologías Pediátricas (SEHOP), Oncología Pediátrica, Departamento de Hematología y Trasplante de Células Madre Hematopoyéticas, Hospital Universitario Infantil Niño Jesús, Madrid, Spain; 6 Sociedad Española de Anatomía Patológica (SEAP), Departamento de Patología, Laboratorio de Dianas Terapéuticas, Hospital Universitario HM Sanchinarro, CIBERONC, Madrid, Spain; 7 Sociedad Española de Oncología Médica (SEOM), Departamento de Oncología Médica, Hospital Universitario La Princesa, Universidad Autónoma de Madrid, Cátedra UAM-Fundación Instituto Roche de Medicina Personalizada de Precisión, Madrid, Spain; 8 Sociedad Española de Anatomía Patológica (SEAP), Departamento de Patología, IIS-Fundación Universitaria Jiménez Díaz, CIBERONC, Madrid, Spain

**Keywords:** Gene fusions, Molecular oncology, Mutations, Neoplasm, Target therapies

## Abstract

The recent identification of rearrangements of neurotrophic tyrosine receptor kinase (*NTRK*) genes and the development of specific fusion protein inhibitors, such as larotrectinib and entrectinib, have revolutionised the diagnostic and clinical management of patients presenting with tumours with these alterations. Tumours that harbour *NTRK* fusions are found in both adults and children; and they are either rare tumours with common *NTRK* fusions that may be diagnostic, or more prevalent tumours with rare *NTRK* fusions. To assess currently available evidence on this matter, three key Spanish medical societies (the Spanish Society of Medical Oncology (SEOM), the Spanish Society of Pathological Anatomy (SEAP), and the Spanish Society of Paediatric Haematology and Oncology (SEHOP) have brought together a group of experts to develop a consensus document that includes guidelines on the diagnostic, clinical, and therapeutic aspects of *NTRK*-fusion tumours. This document also discusses the challenges related to the routine detection of these genetic alterations in a mostly public Health Care System.

## Introduction

The identification of new therapeutic targets and the development of selective tyrosine kinase inhibitors or antagonistic monoclonal antibodies have enriched the therapeutic arsenal against cancer, particularly benefiting subgroups of patients with tumours that harbour specific molecular alterations [1]. The identification of rearrangements of neurotrophic tyrosine receptor kinase (*NTRK*) genes in a wide range of tumours and the development of specific inhibitors of fusion proteins have revolutionised the diagnostic and clinical management of patients who present with tumours with these alterations [1]. The *NTRK* genes encode tropomyosin receptor kinase (Trk) proteins, which play key roles in the development, maintenance, and functioning of neural tissues [2], in addition to a role in the oncogenesis of certain types of tumours [3, 4].

Tumours that harbour *NTRK* fusions are found in both adults and children, and can be classified into two groups [1]. The first group consists of rare tumours with common fusions that are often diagnostic; and the second group comprises more common tumours that rarely harbour *NTRK* fusions (frequencies ranging between 0.1% and 2.0%).

Traditionally, targeted therapies have been developed individually depending on the histological type of the tumour. However, basket trials have shown that the response of some of these inhibitors may be independent of histology [5, 6]. This poses several challenges. One of them is the need to implement comprehensive diagnostic strategies that cover many different types of tumours to benefit small subgroups of patients. The implementation of new diagnostic strategies requires a learning process within hospitals and a balanced use of resources, especially when a high benefit from targeted therapies is expected in a limited number of patients. Three key Spanish medical societies (the Spanish Society of Medical Oncology [SEOM], the Spanish Society of Pathological Anatomy [SEAP], and the Spanish Society of Paediatric Haematology and Oncology [SEHOP], which are responsible for the diagnostic and clinical management of patients with *NTRK-*rearranged tumours, have brought together a group of experts to develop a consensus document that includes guidelines on the diagnostic, clinical, and therapeutic aspects of these tumours. This document also discusses the challenges related to the routine detection of these alterations in a mostly public reimbursement setting.

## Anatomopathological aspects

### Biology of *NTRK*

The *NTRK1*, *NTRK2*, and *NTRK3* genes encode the Trk A, B and C proteins, respectively, that have high affinity towards their ligands, neurotrophins. These ligands and receptors regulate the development, maintenance, and function of neurons. There is a high degree of homology between Trk proteins [7].

*NTRK1*, *NTRK2* or *NTRK3* can be found as oncogenic drivers in a wide range of paediatric and adult tumours. In almost all cases, the 5′ region of a gene that is expressed in the tumour fuses with the 3′ region of one of the *NTRK* genes. The fusion transcript, controlled by the promoter of the 5′ gene, encodes a protein that comprises the amino-terminus of the 5′ gene and the carboxyl-terminal tyrosine kinase domain of the Trk. This results in a constitutively active fusion protein [8]. This constitutive activation leads to an uninterrupted signalling message downstream that acts as a true oncogenic controller. Although fusions can occur in any of the three *NTRK* genes, most of the alterations identified to date involve *NTRK3* or *NTRK1*. The *NTRK* genes show very complex alternative splicing patterns in normal and tumour tissues, which generate multiple types of fusions according to the combination of exons involved in them.

The Trk fusion proteins are often mutually exclusive with other known fusion proteins involving kinases. Specific fusions of the *NTRK* gene are associated with certain tumours (e.g. the *ETV6*–*NTRK3* fusion gene is detected in 90–100% of mammary analogue secretory carcinomas, more than 90% of secretory breast carcinomas, and most cases of infantile fibrosarcoma and congenital mesoblastic nephroma). *NTRK1-3* fusions have been described in infantile fibrosarcoma and mesoblastic nephroma (e.g. *LMNA*–*NTRK1*, *EML4*–*NTRK3*). In some tumours, the proteins encoded by the *NTRK* gene have many different fusion partners. In lung cancer, for example, seven different gene fusions involving the *NTRK1* gene have been described that lead to the constitutive activation of the TrkA tyrosine kinase domain [8]. This suggests that a diagnostic strategy based on the incidence of these fusions and Trk expression patterns in different types of cancer may be the most effective approach to identifying patients whose tumours harbour *NTRK* fusions [7].

Although fusions involving the *NTRK1, NTRK2*, *and NTRK3* genes represent the main mechanism of activation and abnormal expression of Trk proteins, other molecular mechanisms have also been described that have potential impacts on their function. Specifically, overexpression of TrkA and TrkC is a favourable prognostic biomarker in neuroblastoma, while TrkB is often expressed in neuroblastomas with *MYCN* amplification, which per se is an unfavourable biological factor in these patients [9]. Activating splice variants of *NTRK1* have been described in neuroblastoma [10] and have been recognised as having oncogenic capacity.

### Methods of detecting NTRK fusions

The ability to identify *NTRK* fusions has undoubtedly benefited from the wealth of knowledge accumulated in the pathology departments for other treatable rearrangements (*ALK, ROS1*, etc.) [11, 12] The techniques most used for this purpose are immunohistochemistry (IHC), fluorescence in situ hybridization (FISH), next*-*generation sequencing (NGS), and reverse transcription polymerase chain reaction (RT-PCR) [13]. In the case of *NTRK* fusions, the European Society for Medical Oncology (ESMO) issued a clinical practice guideline recommending first screening with IHC and then confirming all positive cases with a second technique (mainly NGS but also FISH in some specific situations; see below) [13]. As discussed in other SEAP–SEOM consensus documents, obtaining a specimen of sufficient quality and quantity to measure the biomarkers that need to be studied in a particular patient should be a responsibility shared by the entire tumour board [14]. For this purpose, it is important that the professionals involved have sufficient knowledge of the advantages and disadvantages of each technology (Table 1) [8, 14, 15]. It would be wise to establish automated and routine channels that would provide a solution when one of the testing techniques fails or is incomplete [14]. To minimise this risk, it is important to keep in mind the preanalytical and sample prioritisation requirements suggested in previous publications [14, 16].

**Table 1:** Advantages and disadvantages of the main approaches to studying *NTRK* fusions

	IHC	FISH	NGS
Advantages	High sensitivity Inexpensive and accessible Fast	High sensitivity and specificity Inexpensive and accessible Fast	High sensitivity and specificity Simultaneous study of other targets
Disadvantages	Specificity unknown Non-standardised interpretation	Three individual FISH tests must be performed Non-standardised interpretation	Expensive and limited access Reduced sensitivity for DNA panels Longer response time

*IHC* immunohistochemistry, *FISH* fluorescence in situ hybridization, *NGS* next-generation sequencing (massive parallel sequencing)

#### Immunohistochemistry

**Fig. 1:**
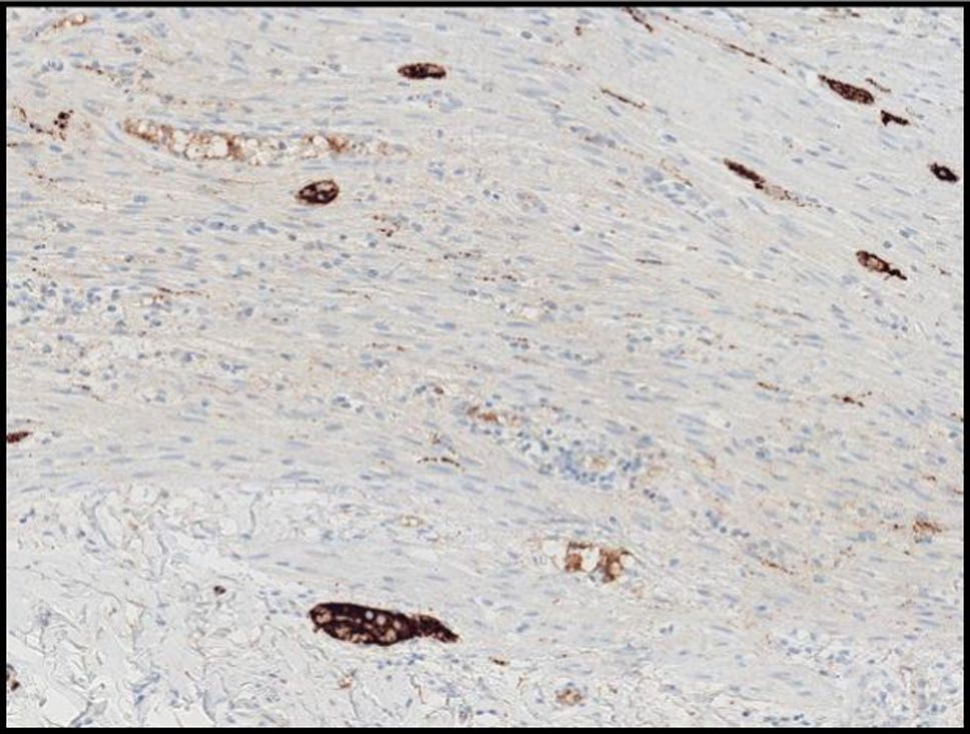
The appendix serves as both a positive and a negative control for the IHC of Trks. The positivity of the neural structures of the appendix wall ensures the correct functioning of the analytical step of the IHC (clone EPR17341, Window, × 400). *IHC*: immunohistochemistry

**Fig. 2:**
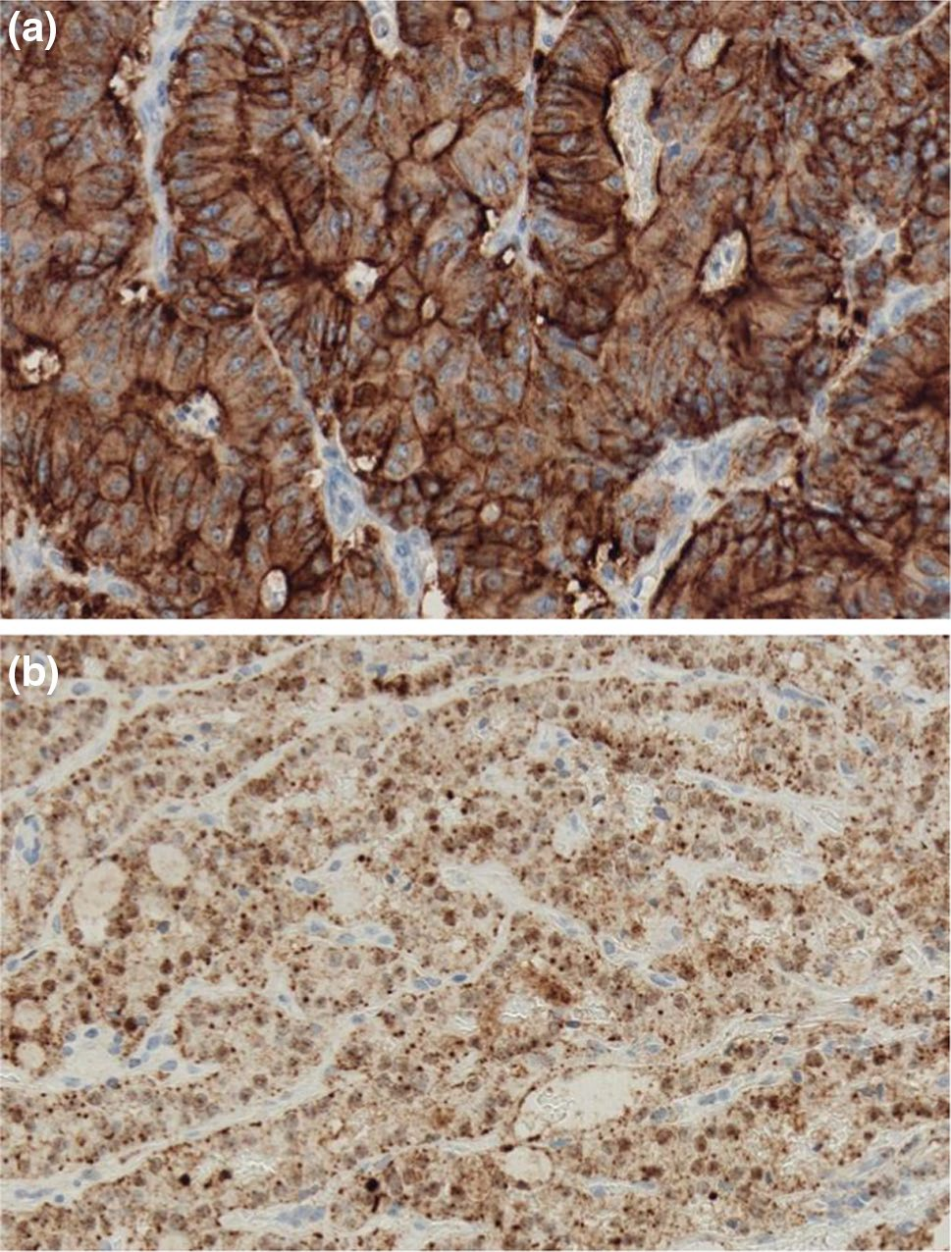
Positive neoplasias can have cytoplasmic staining (**a** adenocarcinoma of the colon, *TPM3*–*NTRK1*, clone EPR17341, Window, × 400) or nuclear staining (**b** papillary thyroid cancer *ETV6*–*NTRK3*, clone EPR17341, Window, × 400)

When IHC is used as a screening method, maximum sensitivity must be achieved, which is particularly important in the case of *NTRK* fusions due to their low prevalence, because once a report of IHC negativity is issued, it is unlikely that this patient will undergo another technique to rule out a rearranged *NTRK*. Therefore, considerations should include (i) choosing an antibody with the greatest accumulated evidence in the literature to identify the overexpression of all three *NTRK* genes (EPR17341) [17, 18], and (ii) a positive control in all slides to ensure the proper interpretation of the result. The most commonly used positive control tissue is from the appendix [18, 19]. The neural structures of the wall should be positive, in contrast to the rest of the tissue (which is completely blue) (Fig. 1). Positivity can be cytoplasmic, nuclear, or mixed (Fig. 2a, b). The cytoplasmic staining is granular and homogeneous throughout but may be stronger in the membrane. Nuclear positivity has been described in about half of patients with the *ETV6*–*NTRK3* fusion [15, 20, 21].

Although there is no universally accepted results interpretation system, some considerations may be useful for establishing the level of IHC positivity that would trigger a confirmatory technique: (i) although it has been suggested that 1% positive cells is sufficient for an IHC diagnosis of positivity [21], most positive cases have staining in 50% or more of the neoplastic cells, with intensities of at least 2 + (on a scale of 0 to 3 +). Lower percentages or intensities seem to be linked more to preanalytical or analytical technical difficulties than to the biology of the neoplasia in question; (ii) structures with neural and smooth muscle differentiation, as well as their neoplastic counterparts, show intrinsic expression of *NTRK* that is not caused by an *NTRK* fusion. Therefore, in all these situations, the value of IHC as a screening method is very limited [15].

#### Fluorescence in situ hybridization

There are three main purposes for FISH in the *NTRK* study algorithm [8, 15]: (i) to confirm or rule out *NTRK* fusions in cases with IHC positivity; this is done by performing three FISH procedures, one each for *NTRK1*, *NTRK2*, and *NTRK3;* (ii) to confirm nuclear IHC positivity for *NTRK3*; and (iii) to confirm *NTRK* fusion in a neoplasm whose histology predicts that type of fusion (e.g. a FISH for *NTRK3* in a secretory breast carcinoma).

The use of dual-colour break-apart probes is recommended. Although the interpretation of the results is not standardised, the general recommendations could be very similar for *ALK* FISH interpretation [12]. At least 50 cells must be counted, with a cut-off for positive nuclei of 15–20% (separate red and green signals and isolated red signals) [22, 23]. Some commercial probes may not detect certain rearrangements due to their design, but there are no published data on this problem yet.

#### Massive parallel sequencing or next-generation sequence

For the correct interpretation of NGS results, several issues should be taken into account [7, 13, 24, 25]: (i) the three *NTRK* fusions are mutually exclusive and do not usually appear together with the main treatable alterations in most neoplasias; (ii) not all sequencing panels include all three genes, and the number of fusion pairs that theoretically can be detected is variable. These two parameters do not necessarily correlate with the number of genes in the panel. Therefore, it is necessary to know the “width” of the panel being used (not only the number of *NTRK* genes but also the number of fusion pairs); (iii) RNA panels have shown better sensitivity than DNA panels. Therefore, the absence of an *NTRK* fusion as shown by a DNA panel should be completely ruled out by a second, confirmatory technique, especially if IHC positivity is unquestionable. Likewise, if the RNA is not of a high enough quality to inform that part of the NGS panel, it is essential to confirm or rule out the presence of *NTRK* fusions (and other treatable rearrangements) through two alternatives: (i) repeating the test in another block of paraffin, either from the same biopsy or surgical specimen or from another, anterior or posterior sample (e.g. rebiopsy); and (ii) using a confirmatory technique.

#### Reverse transcription polymerase chain reaction

The use of RT-PCR for the detection of *NTRK* fusions in RNA has been described in thyroid neoplasms [26], glioblastomas [27], congenital fibrosarcomas [28], and secretory carcinomas of the salivary gland [29] and of the breast [30]. As in other fusion study scenarios, the sensitivity of this technique, the need to have foreknowledge of the gene that makes up one half of the fusion protein, the complexity and variability of the rearrangements that have been described, and the limited preservation of RNA in paraffinised tissue, all suggest that the usefulness of RT-PCR in clinical practice may be limited. There are other alternatives for studying RNA, such as nCounter technology, which is still being developed.

### Organisational aspects

#### Workflow

When a diagnostic test is used in patient populations with low-prevalence molecular alterations, efficiency and costs should be analysed. In this regard, pan-Trk IHC is a reliable and efficient screening method for the detection of *NTRK* fusions. In cases that demonstrate any degree of fusion protein expression by IHC, the alteration should be confirmed by a technique based on DNA or RNA, including FISH, NGS, or PCR. In those centres in which the molecular diagnosis is based on NGS techniques, the use of panels that include the genes of the *NTRK* family and can, therefore, detect their fusions is recommended.

#### Optimal biological specimens

Obtaining sufficient specimens of optimal quality for the study of biomarkers in a particular patient should be a responsibility shared by the entire multidisciplinary committee, as has been commented in other SEAP–SEOM consensus documents [14, 16]. It is important that all involved have sufficient knowledge of the advantages and disadvantages of each technique for studying *NTRK* alterations (Table 1) [8, 15]. It would be helpful to establish automated and routine channels for the study of *NTRK* alterations when diagnosing certain tumour types or according to the clinicopathological characteristics, to ensure an adequate response time. The fundamental parameters to consider for a successful biomarker study are the tumour percentage and the number of tumour cells in the specimen, as well as their preanalytical conditions, as suggested in previous publications [14, 16].

The first step to consider for obtaining an adequate specimen is the time taken between removing the sample from the patient and its fixation, named as cold ischemia time. The general optimal specimen requirements are storage in a 10% buffered formalin solution for 6–12 h for small biopsies and 24–48 h for surgical resections [31] and the presence of at least 50 viable cells for IHC or FISH tests [13]. For PCR and NGS techniques, a minimum of 5% and 20–30% tumour cells are recommended, respectively [32].

#### Quality control

Ensuring the quality of diagnostic techniques is necessary and should be incorporated into the quality control plan of the laboratory or service that performs the tests. In Spain, it is recommended that laboratories have ISO 9001 certification and that the different tests be accredited by the UNE-EN ISO 15,189 standard, which the pathology and molecular diagnostic laboratories have begun to apply and which is evaluated by the Spanish National Accreditation and Certification Entity (ENAC) [33]. The quality control policy should be extended to include (i) personnel involved (technicians, biologists, pathologists, etc.) and their training, experience, and use of standardised work procedures (SWP); (ii) the use of European Conformity (CE)-certified equipment that is properly calibrated; and (iii) the use of validated reagents [34]. In addition, the laboratory should (i) include positive and negative internal controls associated with each test (e.g. brain parenchyma or organs with nerve plexuses); (ii) participate in external quality control programmes (SEAP, EMQN, UK-NEQAS); and (iii) monitor the results to verify that the percentage of mutations found corresponds to the frequency described in the literature according to the type of specimen analysed.

#### Results reporting

The results report must also meet some quality parameters, such as (i) a recommended response time of 7–10 working days; (ii) compliance with the quality control policy described above and (iii) inclusion of the following information: identity of the patient and the person who ordered the test, pathological diagnosis, type of specimen submitted, time of collection (e.g. diagnosis, relapse, or progression), date on which the specimen was collected, collection medium (e.g. fresh, frozen, or paraffin-embedded), anatomical origin, order date, specimen receipt date, date on which the results were issued, test method used, description of the detectable alterations, and potential limitations of the assay. In the case of commercial kits, the commercial name, the batch number, whether it is an approved in vitro diagnostics product, description of the quality of the sample (percentage of cancer cells, whether the sample was enriched by micro- or macrodissection, DNA concentration and purity), comments about the adequacy of the sample, test results defining the type of molecular abnormality detected or the absence of molecular abnormalities, identity of the professional(s) responsible for the test, and, finally and optionally, the name of the laboratory supervisor, should be recorded. Any additional information or comments of interest, accreditation, certification, or participation in quality programmes should also be described.

## Clinical aspects

*NTRK* genes can form parts of constitutively active fusion proteins that lead to the development of multiple types of tumours. Currently, there is a great deal of scientific evidence for the efficacy of Trk inhibitors in the control of the disease in these patients.

### Larotrectinib

Larotrectinib is a selective inhibitor of the Trk proteins (including TrkA, TrkB, and TrkC) approved by the American Food and Drug Administration (FDA) in 2018 for the treatment of adult and paediatric patients with any advanced cancer with a Trk fusion protein after progression following standard treatment or who lack a satisfactory alternative treatment [35]. Likewise, in September 2019, the European Medicines Agency (EMA) approved its use as treatment in adult and paediatric patients with locally advanced, metastatic solid tumours and an *NTRK* fusion, or in those in for whom surgical treatment involves severe morbidity and the patient does not have other satisfactory therapeutic options [36].

The efficacy and safety of larotrectinib have been studied in three multicentre, open-label, single-arm clinical trials in adult and paediatric cancer patients (Phase 1 adult NCT02122913, Phase 1/2 paediatric NCT02637687 “SCOUT”, Phase 2 “in basket” in adolescents and adults NCT02576431 “NAVIGATE”) [37]. In the latest joint analysis of these studies, presented in September 2020, the activity of the drug was analysed in 175 patients who had progressed following a previous standard treatment or for whom effective therapies were not available. The main objective of the analysis was the objective response rate (ORR), which was 78% (95% CI 71–84) regardless of histology, age, and type of *NTRK* fusion (Table 2). In the cohort of adult patients, the ORR was 71% (95% CI 62–79), compared to 92% (95% CI 81–97) in the paediatric cohort. In the general population, after a median follow-up of 12.9 and 11.1 months, the median duration of response (DR) was not reached, and the median progression-free survival (PFS) was 36.8 months (95% CI 25.7-not estimated [NE]). The percentage of patients alive at 1 year was 90% (95% CI 85–95), and at 2 years it was 83% (95% CI 75–90). With a median follow-up of 15.3 months, the median overall survival (OS) had not been reached [38, 39].

**Table 2:** Efficacy of larotrectinib as a function of tumour type*

Type of tumour	*N* = 153 evaluable (%)	Response *n* = 121, 79%
Childhood fibrosarcoma	29 (18)	27 (96)
GIST	4 (3)	4 (100)
Other soft-tissue sarcomas	36 (23)	29 (81)
Thyroid	26 (16)	19 (79)
Salivary glands	21 (13)	18 (90)
Lung	12 (8)	9 (75)
Colon	8 (5)	4 (50)
Melanoma	7 (4)	3 (43)
Breast	5 (3)	3 (75)
Bone sarcoma	2 (1)	1 (50)
Cholangiocarcinoma	2 (1)	1 (50)
Pancreas	2 (1)	1 (50)
Congenital mesoblastic nephroma	1 (< 1)	1 (100, 3–100)
Appendix	1 (< 1)	0 (not calculable)
Hepatocellular	1 (< 1%)	0 (not calculable)
Prostate	1 (< 1%)	0 (not calculable)
Unknown	1 (< 1%)	1 (100%, 3–100)

*GIST* gastrointestinal stromal tumour, *STS* soft-tissue sarcoma

^*^Data cut-off: 19 February 2019

Larotrectinib was designed to have low central nervous system (CNS) penetration, reducing the potential for on-target toxicity through the inhibition of TRKs in the brain. During the clinical development programme of larotrectinib, baseline brain imaging in asymptomatic patients was not required and only 13 (8%) of 159 adult and paediatric patients had baseline CNS metastases. In a *post hoc* exploratory analysis of evaluable patients with brain metastases, 9 of 12 patients (75%) achieved ORR. Only three of 12 patients with evaluable intracranial disease had measurable intracranial disease at baseline. In these patients, best intracranial responses included one complete response, one partial response and one stable disease [38, 40].

The safety of larotrectinib was analysed in 260 patients, and the most frequent adverse events (AEs) were asthenia, cough, elevated liver enzymes, constipation, diarrhoea, dizziness, and anaemia, mainly grade 1–2. Some 16% of patients had grade 3–4 toxicity related to treatment, and it was necessary to discontinue treatment in 2% of patients (6 of 279). The most frequent grade 3–4 related AEs were elevation of alanine aminotransferase (4%), neutropenia (3%), and anaemia (2%) [38, 39].

To study the efficacy of the treatment in the adult population, an analysis of those patients older than 18 years treated with 100 mg larotrectinib every 12 h was performed. With a July 2019 data cut-off, 116 patients and 17 tumour types were registered. The most frequent histological subtypes were thyroid tumours (22%), salivary gland tumours (19%), soft-tissue sarcomas (16%), lung cancer (12%), colon cancer (7%), melanoma (5%), breast cancer (5%) and gastrointestinal stromal tumours (3%). The most frequent fusion transcripts were *NTRK3* (54%) and *NTRK1* (43%), and only 3% had *NTRK2*. An ORR of 71% (95% CI 62–79) was observed, independent of tumour type. With a median follow-up of 17.4 months, the DR was 35.2 months (21.6-NE), and 61% of the patients who responded were progression-free at 1 year of treatment. With a median follow-up of 15.8 months, the median PFS was 25.8 months (15.2-NE), with the median OS not being reached (36.5-NE), and 87% of patients were alive at 1 year [41].

In the analysis of the efficacy of larotrectinib in paediatric patients, as of 30 July 2018, 38 patients under 18 years of age with solid relapsing or locally advanced tumours with *NTRK* fusions included in the Phase 1/2 study (NCT02637687) or in the Phase 2 study (NCT02576431) were reviewed [42]. The included patients had infantile fibrosarcoma (48%), other soft-tissue sarcomas (40%), papillary thyroid cancer (6%), gastrointestinal stromal tumour (2%), melanoma (2%) or mesoblastic nephroma (2%). The recommended dose was 100 mg/m^2^ twice daily orally, equivalent to 173% of the recommended dose in adults adjusted for body surface area [43]. Among the 34 evaluable patients, the ORR was 94% (12 of 34 complete responses, 18 of 34 confirmed partial responses, and 2 of 34 partial responses pending confirmation). The DR ranged from 6.0 to 26.7 months and was greater than 1 year in 84% of patients [42].

### Entrectinib

Entrectinib is a multikinase inhibitor of the TrkA, TrkB, TrkC, ROS1, and ALK proteins that was approved by the FDA and the EMA in August 2019 and May 2020, respectively, for the treatment of patients older than 12 years with advanced solid tumours with *NTRK* fusions who have progressed following standard treatment, who have not previously received Trk inhibitors, and for whom other appropriate treatments are not available [44, 45].

Three clinical trials have studied the activity of entrectinib in the adult population with tumours harbouring *NTRK* fusions: two Phase 1 (ALKA-372-001 and STARTRK-1 [NCT02097810]) and a "basket" Phase 2 trial still ongoing (STARTRK-2 [NCT02568267]) [46]. Patients who had progressed following standard treatment or for whom this was not possible due to high morbidity from localised disease were included, and 94% of patients received a dose of 600 mg every 24 h. The most frequently represented tumours were sarcoma (24%), lung cancer (19%), salivary gland tumour (13%), breast cancer (11%), thyroid cancer (9%), and colorectal cancer (7%). In the analysis of the first 54 adults included, an ORR of 59% (95% CI 45–72), a DR of 12.9 months (95% CI 7.9-NE), a PFS of 11.2 months (8.0–14.9), and an OS of 23.9 months (95% CI 16.8-NE) were observed.

Entrectinib was specifically designed to cross the blood–brain barrier. Patients with brain metastases were enrolled if they had previous treatment resulting in control of symptoms or were asymptomatic. Patients requiring steroids for their brain metastases could continue their treatment, but they must have received stable or decreasing doses for at least 2 weeks before the start of entrectinib treatment. According to a blinded independent central review assessment, a total of 22% of the patients had metastases in the CNS, among whom entrectinib also showed activity at the CNS level, with an ORR and intracranial ORR of 58% (95% CI 28–85) and 55% (95% CI 23–83), respectively [47, 48]. Seven patients had previously received radiotherapy to the brain. In an update of these data that included 74 evaluable patients, these results were confirmed: the ORR was 64% (52–74), DR was 12.9 months (9.3-NE), PFS was 11.2 months (8.0–15.7), and OS was 23.9 months (16.0-NE) [49] (Table 3). In the safety analysis, 355 patients were evaluated, including data from the Phase 1 study in the paediatric population STARTRK-NG. Entrectinib performed similar to larotrectinib, with a majority of AEs being mild (grade 1–2). The most frequent AEs were anaemia, weight gain, dyspnoea, and asthenia. The most frequently observed grade 3–4 AEs were weight gain (10%) and anaemia (12%). Severe AEs were described in 10% of patients, the most frequent being neurological (3, 4%), and in three patients, treatment was suspended for this reason [46]. The typical class side effects of Trk inhibitors are weight gain, observed in 53% of treated patients; dizziness, which may or may not be associated with ataxia, in 41%; and pain after discontinuing the Trk inhibitor, in 35% of patients. These side effects are manageable with modification of the drug dose or with pharmacological intervention [50].

**Table 3:** Efficacy of entrectinib as a function of tumour type*

Type of tumour	*N* = 74 evaluable (%)	Response *n* = 47, 64%
Breast	6 (8)	5 (83%)
Colorectal	7 (10)	2 (29%)
MASC	13 (18)	12 (92%)
NSCLC	13 (18)	9 (69%)
Neuroendocrine	4 (5)	2 (50%)
Other**	5 (7)	3 (60%)
Pancreas	3 (4)	2 (67%)
Sarcoma	16 (22)	9 (56%)
Thyroid	7 (10)	3 (43%)

*NSCLC* non-small-cell lung cancer, *MASC* mammary analogue secretory carcinoma

*Data cut-off: 31 October 2018

**Cholangiocarcinoma, gastrointestinal, gynaecological, neuroblastoma

In the paediatric clinical trial phase 1/1b of entrectinib (NCT02650401), as of 1 July 2019, a total of 35 patients under 20 years of age with solid tumours in relapse, with a median age of 7 years (range 5 months–20 years), were included [51], of whom 11 had *NTRK* fusions (six high-grade gliomas, two infantile fibrosarcomas, one medulloblastoma, one CNS embryonal tumour and one melanoma). Another eight patients had *ROS1* (n = 4) and *ALK* fusions (*n* = 4). The recommended dose was 550 mg/m^2^ once daily orally or 400 mg/m^2^ in patients unable to swallow intact capsules. The safety profile showed no differences from what had been observed in the adult population, most AEs being mild (grade 1–2), mainly at the haematological and digestive levels. Similar to that observed in the adult population, 7 (21%) patients had grade 1–2 neurological symptoms, including drowsiness, paraesthesia, or ataxia. Of the paediatric patients, 3 (9%) discontinued treatment due to AEs, and 11 (32%) had to lower the treatment dose due to AEs. Only patients with *NTRK, ROS1*, or *ALK* fusions responded to treatment. The ORR of the 11 patients with tumours with *NTRK* fusions was 73% (5 of 8 complete responses and 3 of 8 confirmed partial responses).

### New generation inhibitors

Despite the marked efficacy of Trk inhibitors and, in many cases, the long-lasting response, resistance is common. This can occur through the development of mutations of the *NTRK* gene, mutations of MAPK pathway genes such as *BRAF* (V600E) and *KRAS* (G12D), and the amplification of *MET* [1, 52]. However, second-generation Trk inhibitors have been developed, such as selitrectinib and repotrectinib, which have shown activity in these patients [1, 52, 53].

Selitrectinib is a pan-Trk inhibitor with minimal activity against other kinases. It is effective in the treatment of tumours with *NTRK* fusions that have developed resistance to first-generation Trk inhibitors in the form of a secondary point mutation in the kinase domain [53]. Two ongoing phase 1/2 trials are studying the safety and efficacy of selitrectinib in the adult and paediatric populations (NCT03215511, EudraCT 2017-004246-20).

Repotrectinib is a protein kinase inhibitor derived from the *ROS1*, *NTRK*, and *ALK* genes that efficiently binds to the proteins in its active kinase conformation (i.e. the ATP-binding pocket of the kinase) and prevents steric interference resulting from a variety of clinically resistant mutations [54]. It has just received FDA breakthrough designation for *NTRK*-positive patients with advanced solid tumours who have progressed following treatment with at least one prior line of chemotherapy and one or two prior TKIs. This designation is based on findings from the early interim data of the pivotal phase 2 TRIDENT-1 study, which has shown an ORR of 50% in 3 of 6 patients with *NTRK*-positive, TKI-pretreated advanced solid tumours. Currently, there are ongoing trials evaluating the safety and efficacy of the drug in patients older than 12 years (NCT03093116, EudraCT 2016–003616-13; TRIDENT-1), as well as in children under 25 years (NCT04094610).

### Infantile fibrosarcoma

One of the best examples of the activity and potential benefit of therapy with Trk inhibitors is infantile fibrosarcoma, the most common soft-tissue sarcoma in children under 1 year of age. The treatment is eminently surgical, but when it occurs in locally advanced stages or in locations in which surgery can be especially morbid or mutilating, a neoadjuvant chemotherapy, particularly an anthracycline, may be necessary, with consequent short- and long-term side effects and with a response rate of 62%, which means that surgery is not facilitated in a significant percentage of patients [55]. As an example, in the phase 1 clinical trial of larotrectinib, two of the patients achieved a partial response that allowed limb-sparing surgery with negative margins (R0) and absence of viable tumour cells in the specimen, so they could withdraw from treatment, and no relapse beyond 12 months was seen. A third patient also achieved a partial tumour remission of 93%, with incomplete resection (R1) and with the presence of viable cells in the specimen, so the patient continued treatment in the postoperative period, without evidence of relapse 7 months later [56]. Based on these results, treatment strategies for these patients are being designed, even in the first line. Since October 2019, the Phase 2 clinical trial of larotrectinib in neoadjuvant therapy in patients with infantile fibrosarcoma who had not received previous treatment (NCT03834961) has been underway, with the primary objective of determining the ORR in this subgroup of patients.

## Strategies to optimise the detection of *NTRK* fusions in adult and paediatric patients by tumour type

**Fig. 3:**
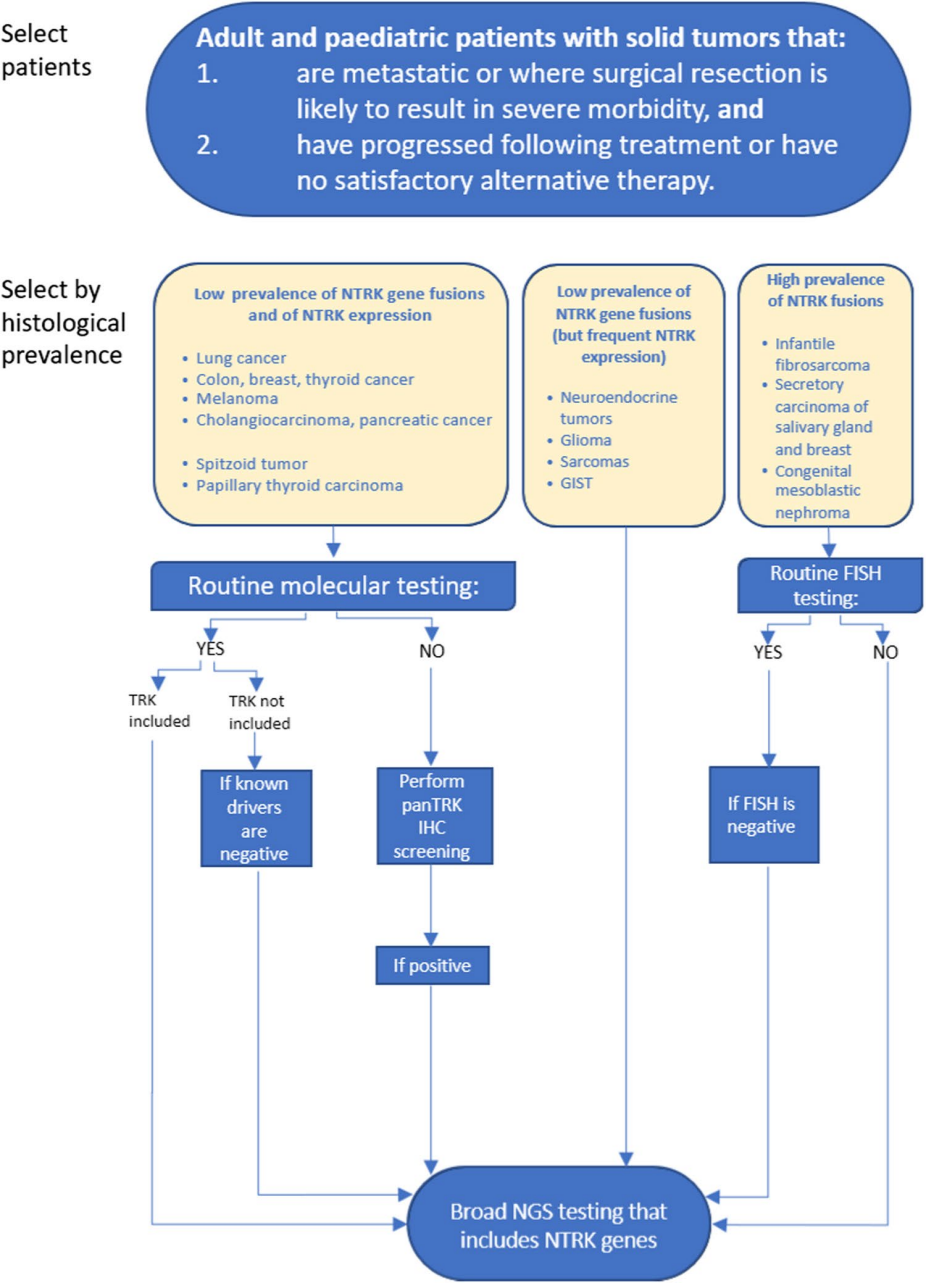
Diagram for the determination of *NTRK* alterations in advanced cancer

Currently, it is recommended to look for *NTRK* fusions in adult patients who have metastatic cancer (or when surgical resection would result in severe morbidity) and who have either progressed following treatment or have no alternative treatment (Fig. 3). In paediatric patients, it is recommended to look for *NTRK* fusions in those whose tumours have a high reported prevalence of such fusions, both at diagnosis and in relapse and regardless of their extent (localised or metastatic) (Table 4). It is recommended to consider searching for *NTRK* fusions in paediatric patients with tumours with a prevalence of *NTRK* fusions lower than those mentioned above at diagnosis: when they are metastatic, when they have a poor prognosis, when surgical resection would result in severe morbidity, and in those who have progressed following standard treatment, who have relapsed, or who do not have an alternative treatment. Searching for *NTRK* fusions is not recommended in patients whose tumours harbour mutually exclusive known mutations with *NTRK* fusions.

**Table 4:** Estimated frequency of *NTRK* gene fusions in different tumour types [20, 59–65]

Adult patient	Paediatric patient
90–100%	91–100%
Mammary analogue secretory carcinoma [66, 67]	Infantile fibrosarcoma [28, 68]
2–15%	92%
Thyroid cancer [69]	Secretory breast carcinoma [70]
4%	83%
Intrahepatic cholangiocarcinoma [71]	Congenital mesoblastic nephroma [68, 72]
< 1–3%	7%
Lung cancer[73]	Non-brainstem high-grade glioma [69]
3%	
Gastrointestinal stromal tumour [74]	
2%	
Colon cancer [75]	
< 1%	
Melanoma [76]	

The evaluation of *NTRK* gene alterations in clinical practice should take into account the expected prevalence according to the histology of the cancer and the availability of standard treatments, as well as the diagnostic techniques that are usually performed in each centre (Table 4) [7, 8, 13, 25]. In cases in which a high prevalence of positive cases for *NTRK* fusion is expected, such as infantile fibrosarcoma, secretory carcinoma of the salivary gland or breast and congenital mesoblastic nephroma, it is recommended to initially perform FISH, if available in the centre, to confirm the expected positivity, or a baseline NGS. If the NGS is negative for *NTRK*, additional tests are recommended. In the cases in which a lower prevalence of *NTRK* fusions is expected, but it is known that there may be an increase in expression by IHC, such as neuroendocrine tumours, gliomas, some sarcomas, or those GISTs in which *KIT* and *PDGFR* are negative and the treatments are therefore limited, it is recommended to perform an initial NGS.

In the remainder of the cases, the diagnostic approach depends on the type of cancer, its genomic profile, and the availability of effective treatments, both in adults and in children. For example, in lung cancer, molecular screening is usually performed. If this takes place, it is important to ensure that *NTRK* is included in the diagnostic test chosen (NGS, FISH, or IHC). In general, if *NTRK* fusion at admission is not determined, and the molecular target test performed does not show alterations in *KRAS*, *NRAS*, *BRAF*, *EGFR*, *ALK*, *ROS1*, *RET* and is considered clinically adequate, it is important still to evaluate *NTRK*s since their fusions are usually exclusive to these targets. *NTRK* evaluation can also be considered to enrich the selection of cases when microsatellite instability is high or deficient in *MLH1*, for example, colon cancer or breast cancer. In general, a two-step procedure may be considered, with an initial screening with IHC for pan-Trk, followed by NGS in positive cases. This may be particularly important in cases where therapeutic alternatives, even first-line alternatives, are unsatisfactory, such as in pancreatic cancer or cholangiocarcinoma [57, 58].

## Conclusions

*NTRK* fusions can be present in a wide variety of tumours, both in adults and in children. In some rare tumours, fusions are found with high frequency, while in more common tumours they are seen in a low percentage of patients, which makes it difficult for oncologists and pathologists specialised in specific areas to gain the necessary experience to identify and diagnose these patients.

In recent years, various Trk inhibitors have been highly effective in the treatment of tumours with *NTRK* fusions, regardless of histology and type of fusion. A limitation of the studies in cancer patients who present *NTRK* fusions is that they are single-arm studies without a standard comparator. The extremely low incidence of those alterations across tumours along with the difficulties to conduct randomised trials in rare diseases and heterogeneous populations are great inconveniences to carry out new studies. However, the activity described with these drugs in this population, without other effective treatment options, led to approval by regulatory agencies in spite of the lack of randomised trials.

Therefore, it is recommended that in the approach to the systemic treatment of adult or paediatric patients with advanced tumours involving aggressive surgeries, *NTRK* fusions be included as one of the biomarkers necessary to adequately guide treatment. It is recommended to always look for *NTRK* fusions in paediatric and adult patients who have tumours with a high reported prevalence of such fusions and to search for *NTRK* fusions in paediatric and adult patients with other tumours with a lower prevalence of fusions if the tumours are metastatic or have a poor prognosis, if surgical resection would result in severe morbidity, and if the patient has progressed following standard treatment or cannot receive an alternative treatment.

A diagnostic strategy for *NTRK* alterations should be defined, following the clinicopathological criteria discussed in this document, while also considering the available resources and the number of cases in each centre and guaranteeing the response time and communication of results, as well as the type of technique for the assessment of *NTRK*. A diagnostic technique based on DNA can be proposed for those tumours with a high frequency of alterations or in which alterations in *NTRK* must be known to make a diagnosis, but IHC is the screening method of choice in most cases. In this scenario, it is necessary to confirm the fusion of the *NTRK* genes by NGS before finalising treatment.

Emerging data suggest that in a significant percentage of cases, secondary events—acquired mutations—confer resistance to first-generation Trk inhibitors, so it is necessary to identify new treatments against these alterations. This situation increases the complexity of the diagnosis, anticipating the need to identify these alterations early on. Therefore, a network of centres of excellence with the availability of adequately funded NGS platforms to ensure equitable access to these complex tests for all patients throughout the national territory must be coordinated by the National Health System. From the SEOM, the SEAP, and the SEHOP, these recommendations are proposed in a context of precision oncology that considers a comprehensive approach to the diagnosis and treatment of cancer, regardless of its histology, based on the knowledge of its molecular alterations.
